# Close Proximity of Left Anterior Descending Artery to the Right Ventricular Lead Apparently Implanted into the Mid-septum

**DOI:** 10.1016/s0972-6292(16)30734-3

**Published:** 2014-03-12

**Authors:** Pavel Osmancik, Petr Stros

**Affiliations:** Cardiocenter, Department of Cardiology, 3rd Medical School, Charles University and University Hospital Kralovske Vinohrady Prague, Czech Republic

**Keywords:** pacing, septal, fluoroscopy, implantation

## Abstract

Right ventricular (RV) mid-septal pacing should have fewer negative effects on left ventricular function compared to apical pacing. However, targeting the mid-septum may be technically challenging since it is usually done with two-dimensional fluoroscopy. The rotation of the heart and various shapes of the RV make it difficult to assess, whether the lead is really anchored in the septum. Many leads, apparently anchored in the septum, are in fact anchored in the anterior wall or anteroseptal groove, and some can get anchored in close proximity to the left anterior descending artery (LAD). We report three cases from our series of 51 patients, in whom the RV lead thought to be implanted in the mid-septum was in fact anchored in close proximity of LAD when assessed using computed tomography.

## Introduction

Pacing the interventricular septum is supposed to produce fewer undesirable effects on left ventricular function. Hence, mid-septal pacing should be preferred over pacing from the right ventricular (RV) apex. The most frequently used method for placing the pacing leads in the mid-septum is by fluoroscopic imaging. An RV lead in the 40º left anterior oblique (LAO40) fluoroscopic view should have a typical orientation, i.e. it should be facing the spine with the angle between the horizontal plane and the lead being between 0 and 60º [1]. We have recently shown that many leads, which fulfill these LAO40 criteria, are in fact anchored outside the septum, either in the anterior wall or anteroseptal groove [2]. In our series of 51 patients, in whom the correct location of the RV lead was assessed using cardiac computed tomography (CT), only 21 (41.2%) patients had leads correctly anchored to the mid-septum. In the remaining 30 (58.8%), leads were in fact anchored to the anterior wall or anteroseptal groove [2]. Moreover, in a detailed analysis of this patient group we found that three patients had the tip of the lead in close proximity (almost touching) to the LAD. Close proximity was defined as a distance between the tip of the lead and the LAD of less than 5 mm. These three patients are the subjects of this report.

## Case reports

### Patient No. 1

**Figure 1:**
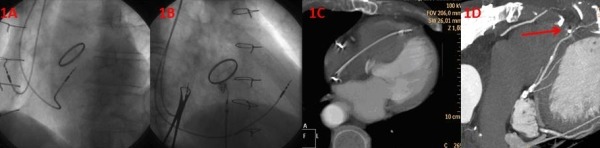
A. Appearance of the right ventricular lead in the LAO40 view. The original fluoroscopic position from LAO 40 view; B. Appearance of the right ventricular lead in the RAO30 view. The original fluoroscopic position from RAO 30 view; C. Cardiac CT showing the actual location of the tip of the lead in the anterior wall (anteroseptal groove); D. Spatial relationship and close proximity of the tip of the RV lead to the left anterior descending artery.

A 78-year-old man had an indication for permanent pacemaker due to a high-degree AV block. Three years prior, he had undergone an aortic valve replacement, mitral valve annuloplasty and coronary artery bypass grafting. The implantation was done through the cephalic vein. First, the RV lead (Vitatron ICQ09B) was inserted into the right ventricle through the tricuspid valve in the posteroanterior (PA) projection. After that, the stylet was manually shaped into a J-shaped curve. The lead was then was advanced into the pulmonary artery in the PA projection Thestylet was then removed, and a short distal angulation was givento the tip of the stylet. The stylet was then reinserted into the lead and, in LAO40 view, the lead was retracted back into the right ventricle until the LAO40 criterion for mid-septal pacing was met, i.e. the angle between the lead and horizontal plane was between 0-60º. The lead was then fixed in this position. The atrial lead was implanted in the appendage. At the end of the procedure, images of lead positions were saved in LAO40, RAO30 and PA view. At implant the parameters were, an RV threshold of 0.6 V at 0.4 mspulse width and QRS amplitude of 10 mV, and the procedure was uncomplicated. At the 6-week follow-up, the pacing parameters were unchanged. A close analysis of a cardiac CT done at this time showed that the RV lead was almost touching the LAD (Figure 1A-1D).

### Patient No. 2

**Figure 2:**
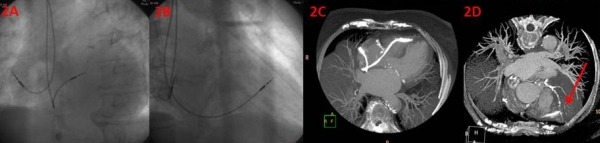
A. Appearance of the right ventricular lead in the LAO40 view. The original fluoroscopic position from LAO 40 view; B. Appearance of the right ventricular lead in the RAO30 view. The original fluoroscopic position from RAO 30 view; C. Cardiac CT showing the actual location of the tip of the lead in the anterior wall (anteroseptal groove); D. Spatial relationship and close proximity of the tip of the RV lead to the left anterior descending artery.

An 81-year-old woman had an indication for a pacemaker implantation for recurrent syncope due to sick sinus syndrome, with sinus pauses (SA arrest) and paroxysms of atrial fibrillation. Pacemaker implant was performed as described above. An active fixation lead (Siello, Biotronik GmbH) was used and the implantation was completed without complication. The RV threshold at implantation was 0.9 V at 0.4 mspulse width and the QRS amplitude was 11 mV; both parameters were unchanged at the 6-week follow-up. A cardiac CT was done at the 4-week follow-upand revealed RV lead was close to the LAD (Figure 2A-2D) as described in the previous patient.

### Patient No. 3

An 80-year-old woman had an indication for a pacemaker implantation for a grade 3 AV block. Pacemaker implant was performed as described above using an active fixation RV lead (Vitatron ICQ09B). At implant the RV threshold was 0.6 V at 0.4 mspulse widthand QRS amplitude was 20 mV. The implantation was uncomplicated and pacing parameters were unchanged at 6-week follow-up. A close analysis of a cardiac CT done at this timeshowed that the tip of the RV lead to be less than 5 mm from the LAD.

**Figure 3:**
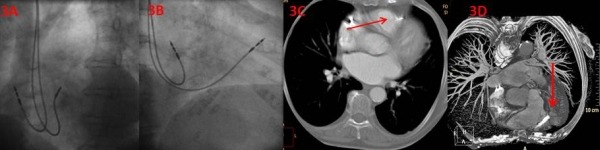
A. Appearance of the right ventricular lead in the LAO40 view. The original fluoroscopic position from LAO 40 view; B. Appearance of the right ventricular lead in the RAO30 view. The original fluoroscopic position from RAO 30 view; C. Cardiac CT showing the actual location of the tip of the lead in the anterior wall (anteroseptal groove); D. Spatial relationship and close proximity of the tip of the RV lead to the left anterior descending artery.

In all three patients, the RV lead had apparently been correctly positioned based on the LAO40 criteria for septal lead placement. However in RAO30 view, the position of the lead tip in all three cases was near the border of the cardiac silhouette (Figure 3A-3D).

## Discussion

The orientation of the RV lead in the LAO40 is the most frequently used criterion for placement in the mid-septum [1].Recently this criterion has been questioned due to its inability to identify the correct mid-septal location of the lead tip. According to a recent study in which the location of the lead was verified using echocardiography, only 54% of leads thathad apparently been implanted in the mid-septum (according to the LAO40 view), were in fact in the mid-septum [3]. In our recently published series using the LAO40 criteria for lead placement, on verification using cardiac CT, it was found that only 41% of leads were correctly placed in the mid-septum [2].

Teh et al. analyzed the relationship between RV lead placement and the LAD using coronary angiography. In their study, the position of the RV lead was assessed using LAO40 criteria. It was found that if the tip of the lead pointed to the right (i.e. towards the spine) in the LAO40, the position was left septum; if the tip of the lead pointed to the left, the position was in the free wall; if the tip of the lead pointed superiorly facing the "screen," the lead was in the anterior wall. In patients with free wall or septal lead locations the lead tips rested some distance from the LAD. However, in patients with the lead in anterior wall, the lead tip was in close proximity to the LAD [4]. Fortunately, this close proximity between the LAD and the lead tip was without clinical consequences. Over and above what was described by Teh et al., though all three patients in this report fulfilled the LAO40 criteria for septalplacement, the leads were not only outside septum but in fact were almost touching the LAD. In all three patients though the RV lead tip was close to the LAD it was completely asymptomatic which is similar to the series published by Teh et al. However, there are case-reports of LAD occlusion caused by a RV defibrillator lead and also an acute occlusion of a left internal mammary artery graft by a RV lead during pacemaker implantation [5,6].

The position of the lead tip in RAO30 is of great importance for confirming the correct placement of a lead tip into the mid-septum. Recently we, along with other authors have shown that if the tip of the RV lead is near the border of the cardiac silhouette in the RAO30 view, then even if the position in the LAO40 indicates mid-septal placement, the tip of the lead is anchored to the anterior wall [2,7]. The true septal position of the lead is associated with the position of the tip of the lead in the middle of cardiac silhouette in RAO30 view [2,7]. Hence, it is suggested that both RAO30 and LAO40 views be used while performing the RV septal lead implant to achieve optimal lead position.

## Conclusion

The three cases in this report demonstrate that the standard LAO40 criterion for proper lead placement in the septum does not guarantee proper mid-septallead position. In fact, leads can easily end up in the anterior wall and dangerously close to the LAD. This potential risk for injury to the LAD needs to be recognized during the implant to prevent possible future complication. For correct mid-septal lead placement, the use of both LAO40 and RAO30 fluoroscopic views are necessary.
